# Immune-Based Prediction of COVID-19 Severity and Chronicity Decoded Using Machine Learning

**DOI:** 10.3389/fimmu.2021.700782

**Published:** 2021-06-28

**Authors:** Bruce K. Patterson, Jose Guevara-Coto, Ram Yogendra, Edgar B. Francisco, Emily Long, Amruta Pise, Hallison Rodrigues, Purvi Parikh, Javier Mora, Rodrigo A. Mora-Rodríguez

**Affiliations:** ^1^ IncellDx Inc, San Carlos, CA, United States; ^2^ Department of Computer Science and Informatics (ECCI), Universidad de Costa Rica, San Jose, Costa Rica; ^3^ Lab of Tumor Chemosensitivity, CIET/DC Lab, Faculty of Microbiology, Universidad de Costa Rica, San Jose, Costa Rica; ^4^ Department of Allergy and Immunology, NYU Langone Tisch Hospital, New York, NY, United States

**Keywords:** COVID-19, PASC, cytokines, chemokines, CCR5

## Abstract

Expression of CCR5 and its cognate ligands have been implicated in COVID-19 pathogenesis, consequently therapeutics directed against CCR5 are being investigated. Here, we explored the role of CCR5 and its ligands across the immunologic spectrum of COVID-19. We used a bioinformatics approach to predict and model the immunologic phases of COVID so that effective treatment strategies can be devised and monitored. We investigated 224 individuals including healthy controls and patients spanning the COVID-19 disease continuum. We assessed the plasma and isolated peripheral blood mononuclear cells (PBMCs) from 29 healthy controls, 26 Mild-Moderate COVID-19 individuals, 48 Severe COVID-19 individuals, and 121 individuals with post-acute sequelae of COVID-19 (PASC) symptoms. Immune subset profiling and a 14-plex cytokine panel were run on all patients from each group. B-cells were significantly elevated compared to healthy control individuals (P<0.001) as was the CD14+, CD16+, CCR5+ monocytic subset (P<0.001). CD4 and CD8 positive T-cells expressing PD-1 as well as T-regulatory cells were significantly lower than healthy controls (P<0.001 and P=0.01 respectively). CCL5/RANTES, IL-2, IL-4, CCL3, IL-6, IL-10, IFN-γ, and VEGF were all significantly elevated compared to healthy controls (all P<0.001). Conversely GM-CSF and CCL4 were in significantly lower levels than healthy controls (P=0.01). Data were further analyzed and the classes were balanced using SMOTE. With a balanced working dataset, we constructed 3 random forest classifiers: a multi-class predictor, a Severe disease group binary classifier and a PASC binary classifier. Models were also analyzed for feature importance to identify relevant cytokines to generate a disease score. Multi-class models generated a score specific for the PASC patients and defined as S1 = (IFN-γ + IL-2)/CCL4-MIP-1β. Second, a score for the Severe COVID-19 patients was defined as S2 = (IL-6+sCD40L/1000 + VEGF/10 + 10*IL-10)/(IL-2 + IL-8). Severe COVID-19 patients are characterized by excessive inflammation and dysregulated T cell activation, recruitment, and counteracting activities. While PASC patients are characterized by a profile able to induce the activation of effector T cells with pro-inflammatory properties and the capacity of generating an effective immune response to eliminate the virus but without the proper recruitment signals to attract activated T cells.

## Introduction

Post-acute sequelae of COVID-19 (PASC) is a group of previously infected individuals who experience a multitude of symptoms from several weeks to months after recovering from their acute illness and presumably months after viral clearance. The prevalence of PASC ranges from 10% to 30% of all individuals infected with SARS-CoV-2 (1). These symptoms include joint pain, muscle aches, fatigue, “brain fog” and others. These symptoms can commonly resemble rheumatic diseases such as rheumatoid arthritis, autoimmune disorders, and others such as fibromyalgia and chronic fatigue syndrome (2). Many of these common disorders are caused by inflammation, hyper- and/or auto-immunity and some such as chronic fatigue are associated with viral persistence after an acute infection with pathogens such as Epstein Barr virus (EBV) and Human Cytomegalovirus (CMV) (3). Previous studies demonstrated that elevations of CCL5/RANTES, IL-6 and to a lesser extent TNF-α were elevated in acute COVID-19 (4). Although patients improved using CCR5 antagonists, the levels of these cytokines decreased but not to normal levels suggesting persistent cytokinemia following discharge from hospitals. In addition, studies including those from our laboratory, have suggested that PASC may be caused by persistent SARS-CoV-2 itself (5). Here, we sought to identify possible immunologic signatures of COVID-19 severity and to determine whether PASC might represent a distinct immunologic condition compared to Mild to Moderate (MM) or Severe COVID-19. Further, we addressed the question whether the immunologic profile represents an immune response indicative of prolonged or chronic antigenic exposure. Using machine learning, we identified algorithms that allowed for accurate determination of PASC and Severe COVID immunotypes. Finally, we present a quantitative immunologic score that could be used to stratify patients to therapy and/or non-subjectively measure response to therapy.

## Materials and Methods

### Patients

**Figure 1:**
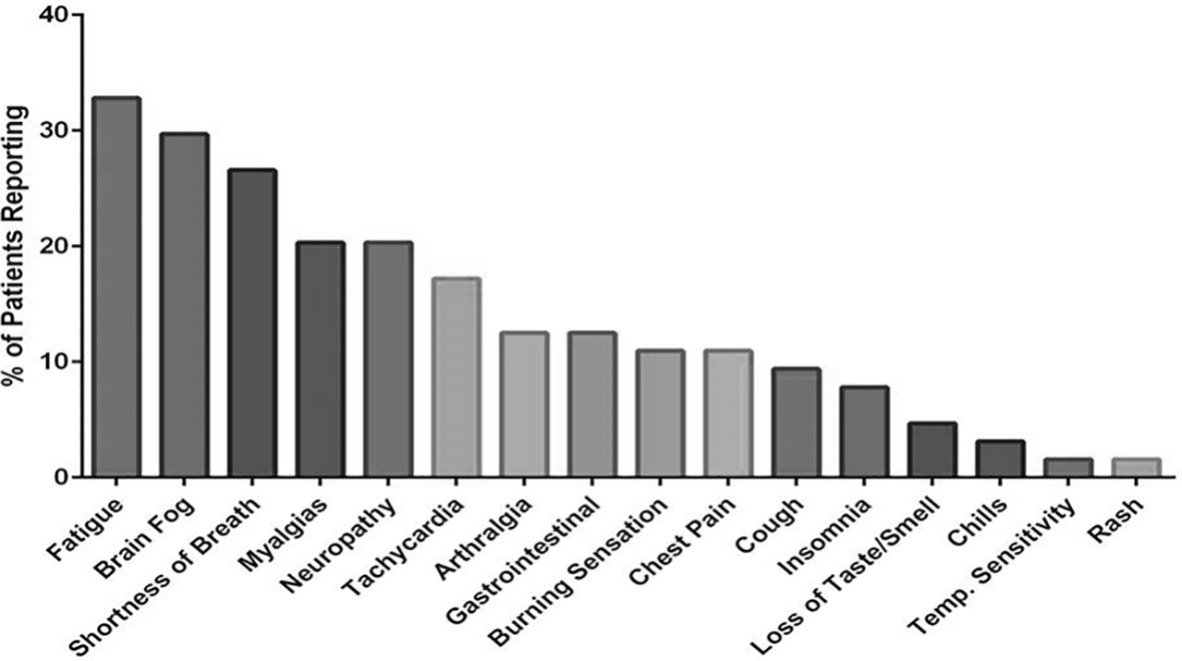
Symptoms reported by PASC patients enrolled in the study.

Following informed consent, whole blood was collected in a 10 mL EDTA tube and a 10 mL plasma preparation tube (PPT). A total of 224 individuals were enrolled in the study consisting of 29 healthy control individuals (negative for both SARS-CoV-2 RNA and SARS-CoV-2 IgM/IgG serology), 26 Mild-Moderate COVID-19 patients, 48 Severe COVID-19 patients and 121 chronic COVID (PASC) individuals (enrolled through the Chronic COVID Treatment Center following informed consent, Protocol CCTC 20-001). PASCs symptoms are listed in **Figure 1**. Study subjects were stratified according to the following criteria.

### Mild

1. Fever, cough, sore throat, malaise, headache, myalgia, nausea, diarrhea, loss of taste and smell
2. No sign of pneumonia on chest imaging (CXR or CT Chest)
3. No shortness of breath or dyspnea

### Moderate:

1. Radiological findings of pneumonia fever and respiratory symptoms
2. Saturation of oxygen (SpO2) ≥ 94% on room air at sea level

### Severe:

1. Saturation of oxygen (SpO2) < 94% on room air at sea level
2. Arterial partial pressure of oxygen (PaO2)/fraction of inspired oxygen (FiO2) < 300mmHG
3. Lung infiltrate > 50% within 24 to 48 hours
4. Heart Rate ≥ 125 bpm
5. Respiratory rate ≥ 30 breaths per minute

### PASC

1. Extending beyond 12 weeks from the initial onset of first symptoms.

### High Parameter Immune Profiling/Flow Cytometry

Peripheral blood mononuclear cells (PBMCs) were isolated from peripheral blood using Lymphoprep density gradient (STEMCELL Technologies, Vancouver, Canada). Aliquots (6) of 5 x 10^5^ cells were frozen in media that contained 90% fetal bovine serum (HyClone, Logan, UT) and 10% dimethyl sulfoxide (Sigma-Aldrich, St. Louis, MO) and stored at -70°C. Cells (5 x 10^5^) were stained and analyzed as previously described (4) using a 14-color antibody cocktail with the volumes indicated (**Supplementary Table 1**). Samples were analyzed on a Beckman Coulter CytoFlex LX 6-laser flow cytometer using Kaluza Analysis Software (Beckman-Coulter, Miami, FL). All statistical analysis was performed using the Mann-Whitney test and a P value ≤0.05 was considered statistically significant.

### Multiplex Cytokine Quantification

Fresh plasma was used for cytokine quantification using a customized 14-plex bead based flow cytometric assay (IncellKINE, IncellDx, Inc) on a CytoFlex flow cytometer as previously described using the following analytes: TNF-α, IL-4, IL-13, IL-2, GM-CSF, sCD40L, CCL5 (RANTES), CCL3 (MIP-1α), IL-6, IL-10, IFN-γ, VEGF, IL-8, and CCL4 (MIP-1β) (4). For each patient sample, 25 µL of plasma was used in each well of a 96-well plate. Samples were analyzed on a Beckman Coulter CytoFlex LX 3-laser flow cytometer using Kaluza Analysis Software (Beckman-Coulter, Miami, FL). All statistical analysis was performed using the Mann-Whitney test and a P value ≤0.05 was considered statistically significant.

### Data Processing

Although we have previously defined healthy, Mild, Moderate, Severe, and PASC patients, for downstream analysis we have divided the patients into 4 classes: Healthy control (healthy patients), Mild-Moderate (including the Mild and Moderate patients), Severe, and PASC. Data was imported and processed using Python 3.8.3, using the *pandas* library (version 1.1.0) (7). and the numeric python module, *numpy* version 1.18.5 (8). Our data consisted of 224 instances representing 4 classes (healthy control, Mild-Moderate, Severe and PASC). The dataset consisted of 16 columns, of which 14 represented the different cytokine/chemokine analytes, one for the patient identifier and one column for the label, or the class to which the patient belonged (healthy control, Mild-Moderate, Severe or PASC).

We identified imbalanced class labels in our dataset, and thus decided to proceed to balance the dataset. In order to adequately do data balancing, it was necessary to separate the data into training, validation and test sets. We used the 60/20/20 schema, with a 20% validation partition to assess model overfitting after training, and 20% of data for class label prediction. Data partitions needed to be implemented in order to ensure that generated samples would be present in the training set only. It is necessary to avoid generated samples in either the validation or test set because their presence in either can lead to overfitting and spurious results. 

### Data Balancing With Synthetic Oversampling of the Minority Class

The 4 classes in our dataset were composed of different numbers of instances. If the variation between the number of classes is large enough, it can lead to a phenomenon identified as class imbalance. The potential existence of class imbalance in our dataset was further supported by the fact that 50% of the dataset, or 121 individuals, were PASCs, while only 26 and 29 were mild-moderate and healthy controls, respectively, and the remaining 48 corresponded to the Severe class. Class imbalance leads to differences in the ratios between classes, for example we identified a 2.5 ratio between PASC and Severe, and a 4-fold ratio between PASC and both Mild-Moderate and Control. These differences in ratios can lead to biased predictions, which are often reflected as poor model performance metrics and generalizations (6, 9, 10). In order to avoid this potential pitfall, balancing methods have been proposed, which include random under-sampling and oversampling methods. However, it has been reported that random under-sampling can lead to information loss (11), whereas basic/randomized oversampling can lead to model overfitting.

Chawla et al. (11) proposed a solution in synthetic oversampling of the minority class. This method, known as SMOTE, uses interpolation between minority class instances to generate new data points to balance the dataset. SMOTE has been used in imbalance, including those of biological context, in conjunction with machine learning models (12). We pipelined SMOTE from the python library imbalanced-learn (13) to balance the training set, which was used in random forest classifier construction.

### Random Forest Classifier

The random forest (RF) classifier is an ensemble method that groups multiple decision trees. Random Forests can be used for both classification and regression problems, as developed in 2001 (14). This method has been used to analyze biological datasets and in biological context knowledge discovery (11, 15, 16). Random forest classifiers possess the advantage of incorporating the option of assessing feature importance, which can be of great importance when undertaking downstream analysis, like assessing the biological significance of a feature, understanding its relevance in a given biological process like immune response, or its potential role as a biomarker (17).

The ability to both be a predictor and identify relevant features makes random forests embedded selection methods. We used the Python’s machine learning library, scikit learn, version 0.24.1 to construct the random forest classifier (18). Additionally, in order to adjust model hyperparameters (number of features, tree depth and number of trees) we used an exhaustive grid search with 10-fold cross validation (CV). It is important to note that variable importance was only implemented to identify significant features and not for dimensionality reduction.

### Defining Precision, Recall and F1 Score for Model Performance

To estimate the random forest classifier performance, we selected three different metrics: precision (equation 1) which is a measure of the percentage of the results that are relevant, recall which measures the total relevant results that are correctly classified by the predictor (equation 2), and the F1 score (equation 3), which is the harmonic mean between these two measures and ranges from 0 to 1. If the F1 score is close to 1, the better the model performs. The F1 score for both false positives (FP) and false negatives (FN) as well as for true positives (TP).

(1) $$Precision=\frac{TruePositive}{TruePositive+FalsePositive}$$

(2) $$Recall=\frac{TruePositive}{TruePositive+FalseNegative}$$

(3) $$F1=\frac{2*Precision*Recall}{Precision+Recall}=\frac{TP}{TP+\frac{1}{2}(FP+FN)}$$

## Results

### Immune Profiling

To determine if immunologic abnormalities remain in PASCs, we performed high parameter immune cell quantification and characterization in a subset of individuals with preserved PBMCs (**Table 1A**). We quantified B-cells, T-cells, and monocytes including subsets and including CD4/CD8 activation and T-cell exhaustion. All T-cells determinations were initially gated on CD3 expression and all monocyte subsets were initially gated on CD45 (**Supplementary Figure 1**). Unlike acute COVID-19 (4), the CD4 and CD8 T-cell populations in PASC were within healthy control limits and there was no evidence of T-cell exhaustion. In fact, CD4 and CD8 positive T-cells expressing PD-1 were significantly lower than normal controls (P<0.001 and P=0.01 respectively). Further, there was a significant decrease in total T regulatory cells compared to healthy control individuals (P<0.001) possibly exacerbating the hyper-immunity in PASC. B-cells were significantly elevated compared to healthy control individuals (P<0.001) as was the CD14+, CD16+, CCR5+ monocytic subset (P<0.001) (**Table 1A**). Interestingly, these two immune cell populations have been shown to be chronically infected by different viruses. B-cells are infected by Epstein-Barr and the CD14+, CD16+, CCR5+ monocytic subset by HIV-1 and by HCV (19).

**Table 1A:** T-, B-cell, and monocyte immunophenotyping.

Average	CD3+%	CD4%	CD8+%	CD4+PD1%	CD4+LAG3%	CD4+CTLA4%	CD4+FoxP3%	CD8+PD1%	CD8+LAG3%	CD8 CTLA4%	CD8+ FoxP3%	CD19%	CD14+CD16-%	CD16+CD14+%	CD16+CD14-%
**Healthy** **Controls**	64.40	53.80	33.83	35.62	0.94	1.51	6.21	43.75	4.35	1.38	0.67	6.04	42.79	9.00	32.67
**Lower CI**	54.39	43.21	27.20	28.36	0.49	0.75	4.54	33.50	2.71	0.74	0.37	5.04	34.41	4.60	25.49
**Upper CI**	74.50	64.57	40.46	42.89	1.39	2.26	7.87	54.01	5.99	2.03	0.97	7.04	51.16	13.41	39.86
**PASC**	48.98	56.18	35.36	17.78	0.72	4.06	2.58	31.99	0.71	3.11	1.01	13.14	19.01	29.3	33.86
**Lower CI**	44.78	52.44	32.56	15.73	0.36	2.32	2.01	29.46	0.55	2.04	0.80	11.72	15.65	25.65	30.28
**Upper CI**	53.18	59.92	38.70	19.83	1.08	5.80	3.15	35.52	0.87	4.18	1.22	14.56	22.37	32.95	37.44

To further characterize the immune response in PASCs, we performed a quantitative, multiplex cytokine/chemokine panel on 29 healthy control individuals to establish the healthy control range of the assay. We then analyzed Mild-Moderate, Severe, and PASCs plasma samples and compared the cytokine/chemokine profiles (**Table 1B**). CCL5/RANTES, IL-2, IL-4, CCL3, IL-6, IL-10, IFN-γ, and VEGF were all significantly elevated compared to healthy controls (all P<0.001). Conversely GM-CSF and CCL4 were in significantly lower levels than healthy controls P=0.005.

**Table 1B:** Cytokine and other soluble factors quantification.

Average (pg/ml)	TFN-α	IL-4	IL-13	IL-2	GM-CSF	sCD40L	CCL5 (RANTE S)	CCL3 (MIP-1α)	IL-6	IL-10	IFN-γ	VEGF	IL-8	CCL4 (MIP-1β)
Healthy Controls	9.09	4.18	3.94	6.17	51.27	7192.39	10781.84	22.82	2.21	0.67	1.94	9.32	16.87	76.84
Lower Cl	7.37	2.17	1.79	5.53	25.72	5148.85	9764.99	13.05	1.65	0.42	0.63	6.36	13.03	61.00
Upper Cl	10.81	6.18	6.09	6.82	76.82	9235.92	11798.68	32.60	2.77	0.92	3.26	12.28	20.72	92.67
PASC	7.72	17.03	4.21	16.16	12.46	18302.41	12505.06	97.81	20.47	12.23	86.60	41.03	35.98	35.10
Mild-Mod	6.82	2.33	2.40	5.90	56.13	10673.72	11627.70	18.75	8.74	0.63	1.15	17.39	17.37	94.40
Severe	5.39	2.39	2.26	5.43	20.31	12306.39	11581.47	16.54	144.15	3.10	2.06	25.52	10.87	64.84

### Construction of a Multi-Class Random Forest Predictor for the Discrimination of the Analytical Groups in the Dataset

We proposed to differentiate the analytical groups (or diseases groups) of the dataset by constructing a multi-class random forest classifier. During the exploratory data analysis phase, we identified that the current dataset presented the characteristic of being imbalanced, with an overrepresentation of the PASC class. This dataset can also be considered medium-sized due to the number of instances. To address these potential pitfalls, and to avoid model overfitting, we implemented a balancing technique as described above. The implementation of SMOTE is thus useful to counter overfitting and to generate new samples from interpolation for the underrepresented or minority classes. By using SMOTE to balance the minority classes to 100% of the PASC class, it resulted in each class having 76 instances in the training set. This represented a 4-fold increase in the healthy control and the Mild-Moderate classes, and a 2.5-fold increase for the Severe class.

**Figure 2:**
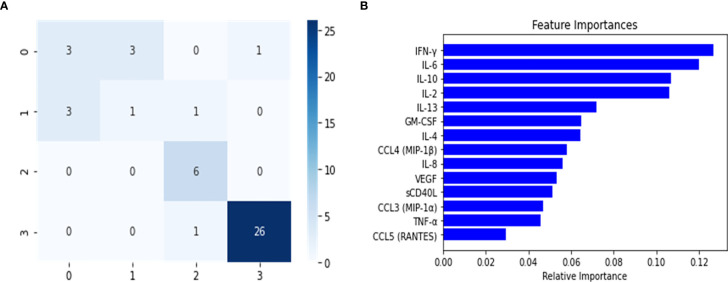
Confusion Matrix **(A)** and Feature importance **(B)** for multi-class classifier using Random Forest predictor. The confusion matrix was calculated from the predictions of the random forest classifier on the test set. The classes were assigned in the following manner: (0) healthy controls, (1) Mild-Moderate, (2) Severe and (3) PASC.

The balanced dataset was used to construct the multi-class RF predictor, which was fine-tuned using the grid-search and cross validation approach. This implementation of grid search and 10-fold CV was used as a fine-tuning approach for this and all subsequently constructed classifiers. The multi-class model was then analyzed for overfitting with the validation set (**Table 2**). During this analysis, we noticed a slight decrease in the model’s predictive performance when discriminating between the healthy control and Mild-Moderate class, however the overall performance in the validation set was high, as seen by the recall (sensitivity) and the f1 score. However, these differences were heavily accentuated in the performance metrics of the test set (**Table 2**). This can be further appreciated in the confusion matrix for the multi-class classifier (**Figure 2**), which demonstrates that in the test split, both the Severe and PASC classes were properly identified but the healthy control and Mild-Moderate classes incurred in multiple misclassifications. Furthermore, when analyzing the feature importance (cytokines) of the dataset, we noticed the differences between variables are of small magnitude, only amplified by the scale of the axis (**Figure 2**), but apart perhaps the difference between IFN-ˠ and CCL5 (RANTES), differences might not be that obvious. Because of these findings, we decided to proceed with the construction of the binary RF classifiers focused on Severe and PASC classes.

**Table 2:** Random forest classifier predictor performances on the validation and test partitions.

Model	Accuracy	Precision	Recall	F1
Multi-class-Val	0.97	0.97	0.92	0.93
PASC-Val	1.00	1.00	1.00	1.00
Severe-Val	0.94	0.95	0.94	0.94
Multi-class-Test	0.8	0.62	0.65	0.63
PASC-Test	0.96	0.95	0.96	0.95
Severe-Test	0.95	0.97	0.93	0.94

The partition is indicated next to the model, either as Val for validation or Test for the test partition. The presented performance metrics were calculated using the classification report and the confusion matrix form sci-kit learn (18).

### Construction of a Binary PASC Random Forest Classifier Allows Identification of Relevant Features for the Development of a Heuristic Score for PASC Patient Identification

**Figure 3:**
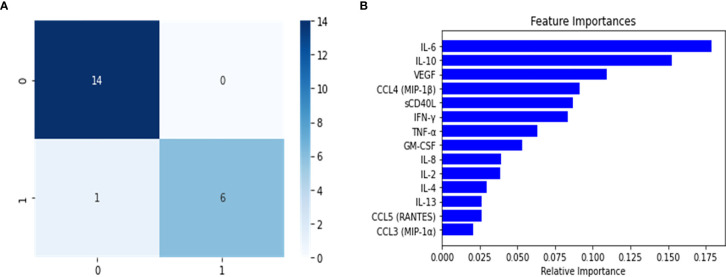
Confusion Matrix **(A)** and feature importance **(B)** for the PASC binary random forest classifier to enable the feature engineering of a score for the identification of PASC patients. The positive class (1) are PASCs while the negative (0) class are the non-PASCs (healthy control, Mild-Moderate-Severe).

After constructing the multi-class predictor, we proceeded with the development of a binary classifier furthering our understanding of the PASC disease group. The PASC class was comprised of long-term disease carriers, and thus the random forest classifier was tasked with separating the long-term carriers from those instances that did not belong to this class, and to identify the cytokines or features that were relevant for the discrimination of the disease groups. To achieve this, we separated the data into two major groups, one that consisted of all the classes (healthy control, Mild-Moderate and Severe) representing non-long term disease carrier groups, and a second with the PASCs. This new dataset was split into 60/20/20 (training/validation/test) and the training set was balanced using SMOTE. The trained classifier was fine-tuned to determine the best hyperparameter combination (tree-depth, feature number, number of trees) using and exhaustive grid search. We then used the model on the validation set in order to detect model overfitting, and did not identify indications of model overfitting (**Table 2**). The model was implemented on the test set, to predict the classes for the instances in this partition. When analyzing the confusion matrix (**Figure 3**), the model’s predictive capabilities seemed very high, with only 2 instances being misclassified, this is further supported by the predictors metrics (**Table 2**), where the F1 score, the balance between precision and recall was 0.95. Additionally, when looking at the variable importance analysis (**Figure 3**), we identified that the top 5 most relevant cytokines were (in order): IFN-ˠ, IL-2, IL-4, IL-10 and GM-CSF. Other relevant identified cytokines include: IL-8, CCL4 (MIP-1β) and CCL3 (MIP-1α).

**Figure 4:**
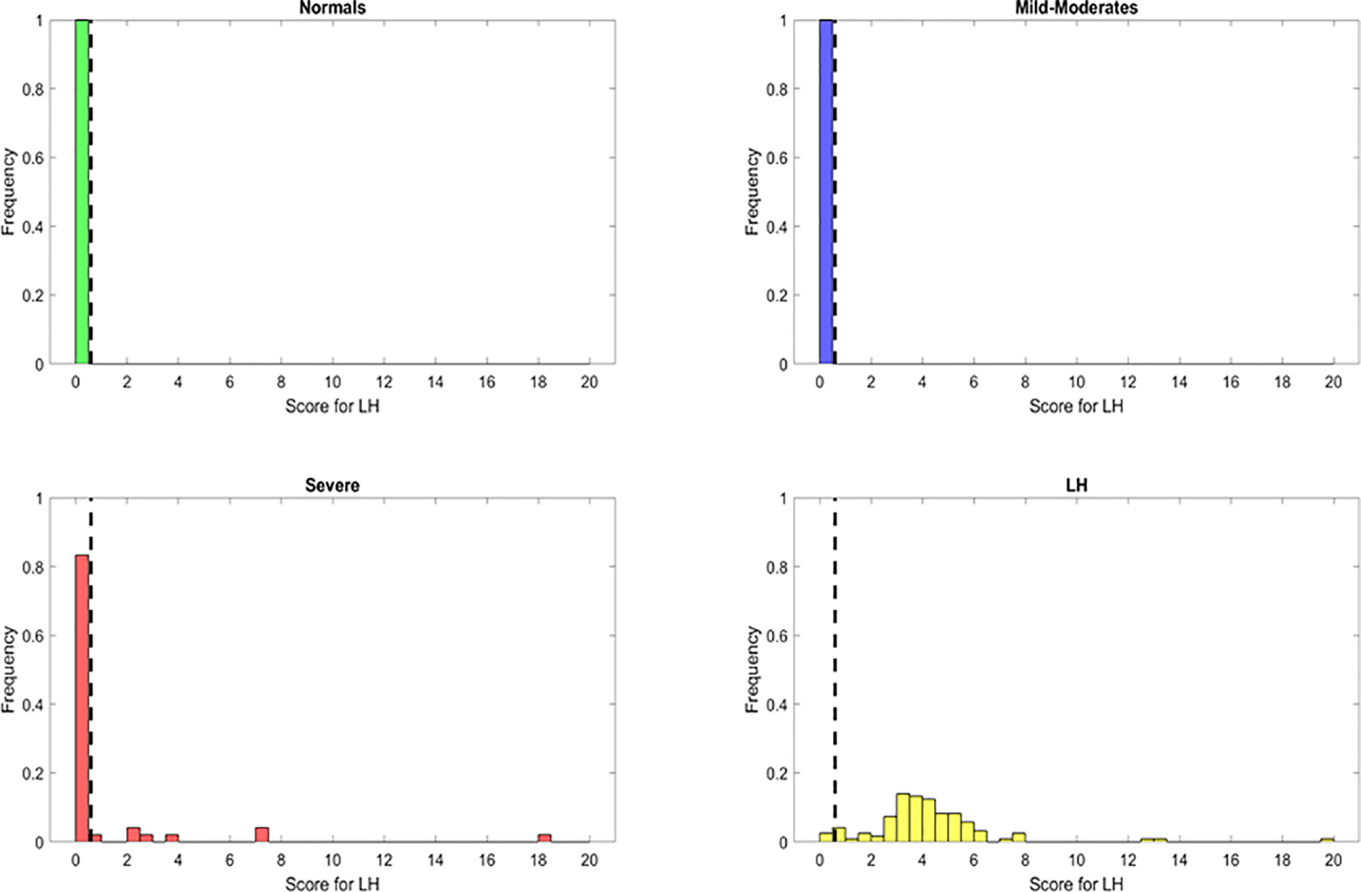
Discrimination ability of the long hauler score for the identification of PASC patients (S1) with reduced or most important features identified using Random Forest classifier. The dots represent the data points, where yellow are PASCs, red-Severe, dark blue-Mild-Moderate and green-healthy control.

The resulting features identified from the variable importance analysis were fundamental for the subsequent development for a novel heuristic that was constructed using feature engineering. Through the use of the score derived from this heuristic, we aimed to simplify our model and gain biological insight about the PASC phenotype. We obtained a “PASC Score” defined as S1 = (IFN-γ + IL-2)/CCL4-MIP-1β (**Figure 4**). Setting an optimized threshold of S1 = 0.5 as a tradeoff of sensitivity and specificity, it was possible to classify the majority of PASCs as such (118/121 with S1 > 0.5) for a sensitivity of 97.5%. No healthy control or MILD-Moderate cases were classified as PASCs (specificity of 100% for healthy control and MILD-Moderates). In contrast, 7/48 Severe cases were classified as PASCs (S1>0.5) for a specificity of 85%, suggesting that these ‘misclassified’ Severe cases could indeed become PASCs.

### Construction of a Binary Random Forest Classifier and Variable Importance Enables the Feature Engineering of a Score for Severe Patient Identification

The random forest classifier for discriminating between Severe and non-Severe individuals was constructed by grouping the balanced healthy control and Mild-Moderate classes into a single group that was labeled as non-Severe. In this dataset, the PASC class was excluded based on the scope of potentially identifying the cytokines that separate the Severe disease group from those that are in a non-Severe state. These non-Severe individuals however, do not belong to a long-term carrier group. In addition, the results from the disease score generated using the important variables allowed us to discriminate the PASCs.

**Figure 5:**
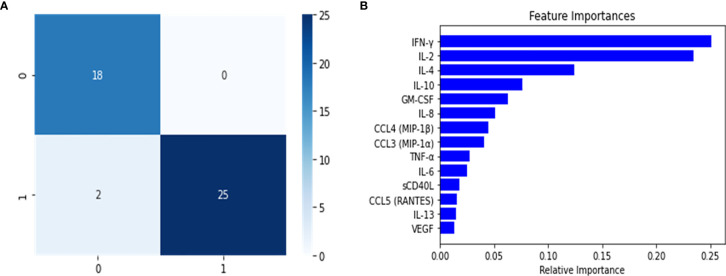
Confusion matrix **(A)** and variable importance **(B)** for the Severe binary classifier constructed using the random forest classifier. The results shown in the confusion matrix were calculated for test split, where 0 represents the grouped Mild-Moderate and healthy control instances, and 1 are the Severe cases. For B, the most significant variables were to calculate a disease group score for Severe patients.

The model was constructed and fine-tuned using the same approach implemented in the multi-class and binary models. The model with the best parameters was then selected to identify model overfitting in the validation set. We were not able to determine any evidence of overfitting, and proceeded to use this model to undertake predictions in the test set. As the confusion matrix for this Severe binary classifier indicates (**Figure 5**), it was possible to discriminate between what we defined as Severe and non-Severe instances. The number of incorrectly classified instances was 1 non-Severe misclassified as Severe (**Figure 5**). The model performed very well, as indicated by its metrics in the test set (**Table 2**). Both precision and recall were high (0.97 and 0.93, respectively, with an F1 score of 0.94). Additionally, as we will report, this model also identified important features (cytokines) that were relevant to discriminate between the disease groups. This information would be useful to develop a heuristic score for the Severe disease group. We also undertook variable importance analysis (**Figure 5**) where we identified as the most relevant features: IL-6, IL-10, VEGF, with IFN-γ, CCL4-MIP-1β and sCD40L being informative to a lesser degree.

**Figure 6:**
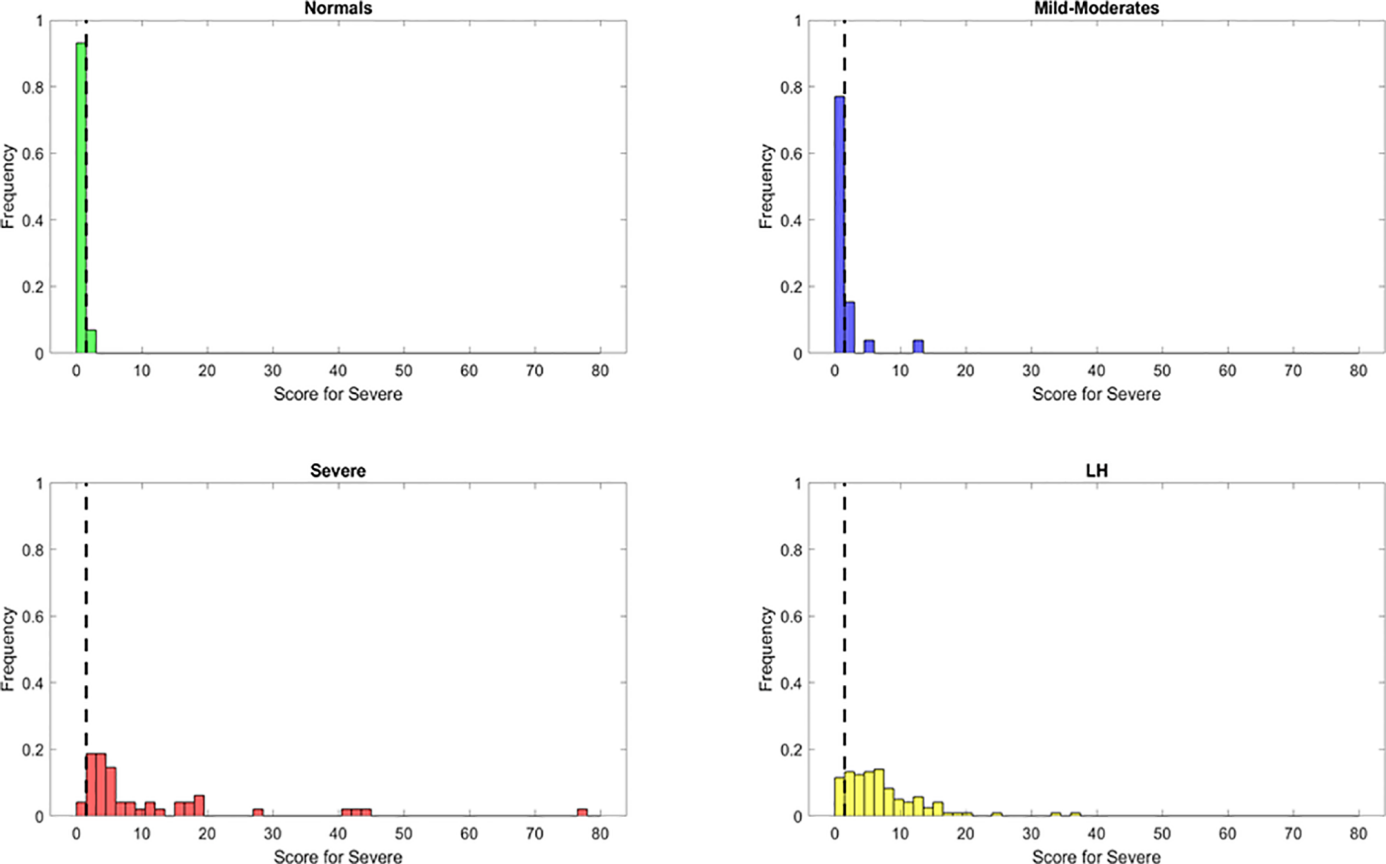
Discrimination ability of the Severe score for the identification of Severe patients (S1) with reduced or most important features identified using Random Forest classifier. The dots represent the data points, where yellow are PASCs, red-Severe, dark blue-Mild-Moderate and green-healthy control.

Using these important features we developed a score to identify patients. Based on the same principle, but using the relevant features from the Severe random forest binary classifier, we engineered a score for the identification of Severe cases. This new score, identified as S2, was calculated as follows: S2 = (IL6+sCD40L/1000+VEGF/10+10*IL10)/(IL2+IL8). Setting an optimized threshold of S2 = 1.5 as a tradeoff between sensitivity and specificity, it was possible to apply the heuristic to classify the majority of Severe as such (46/48 with S2 > 1.5) for a sensitivity of 95.8%. Only 2/29 healthy control and 5/26 MILD-Moderate cases were classified as Severe (specificity of 93% for healthy control and 81% for Mild-Moderates which may be disease status misclassification) (**Figure 6**). However, using this score alone, the original PASCs cannot be separated as most of them will be classified as Severe.

### A Combined Heuristic Enables an Optimal Classification of PASCs and Severe Cases of COVID-19

**Figure 7:**
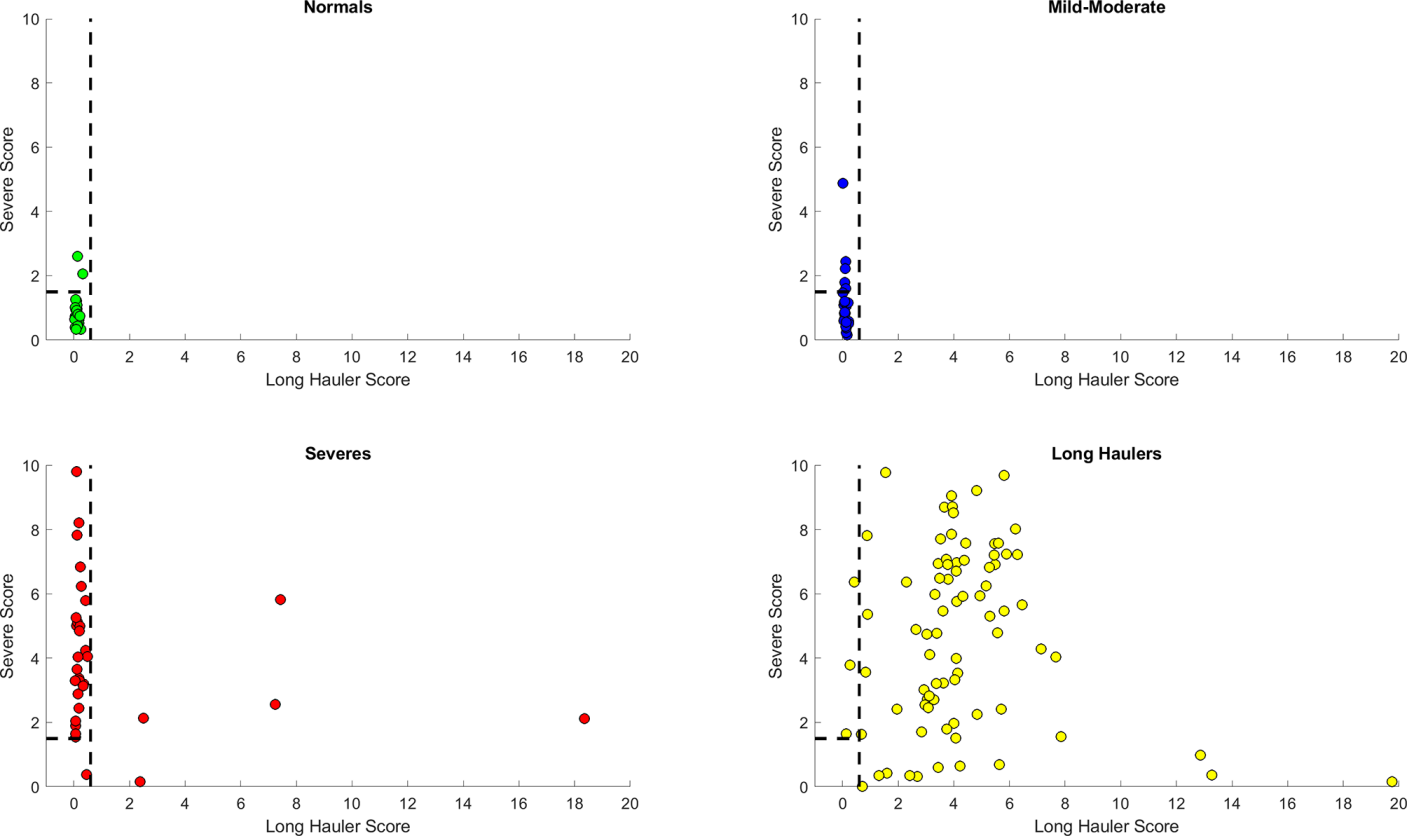
Discrimination ability of the heuristic with both Long Hauler (S1) and Severe (S2) scores. The PASC patients are first identified with an S1>1.5. From those remaining patients, the Severe cases are identified with an S2> 1.5. The dots represent the data points, where yellow are PASCs, red-Severe, dark blue-Mild-Moderate and green-normal.

In order to integrate the PASC and Severe identification, we aimed to develop a combined heuristic using both scores and the optimized thresholds defined above. This heuristic identifies the PASC cases first using the ‘PASC score’ and then identifies the Severe cases from the remaining data points. The graphical representation in **Figure 7** shows a very good separation of the PASC and Severe cases from the healthy control and Mild- Moderates. All PASCs (121) were classified either as PASCs (118) or Severe (3) indicating a sensitivity of 100% to identify pathology. On the other hand, only 1 Severe case was classified as Mild-Moderate, indicating that most Severe cases were classified either as Severe (n=40) or PASC (n=7) indicating a sensitivity of 97.9% to detect pathology. In addition, the presence of those 7 ‘mis-classified’ Severe cases as PASCs suggests that some Severe cases are underway to become PASCs.

Finally, we simplified our prediction model by feature engineering of two classification scores based on the top informative features. First, a “PASC Score” was defined as S1 = (IFN-γ + IL-2)/CCL4-MIP-1β. Second, “Severe Score” was defined as S2 = (IL6+sCD40L/1000+VEGF/10+10*IL10)/(IL2+IL8). Using a combined heuristic to first classify the PASCs (S1>0.4) and second the Severe COVID-19 patients (S2>0), we obtained a sensitivity of 97% for PASCs with a 100% specificity and a sensitivity of 88% for Severe patients with a specificity of 96% (**Figure 7**).

## Discussion

Individuals infected with SARS-CoV-2 exhibit distinct severity patterns which have been associated with different immune activation profiles. Interestingly, in some cases longer times are required to experience full recovery, representing a particular pathological type recently described as long-COVID or PASC.

The scientific evidence generated during the last months strongly supports that the different outcomes on COVID-19 patients are determined by the immune mechanisms activated in response to the viral infection (20).

The immune response to SARS-CoV-2 induces a release of different molecules with inflammatory properties such as cytokines including interleukins and chemokines. This event, known as cytokine storm (20), is an immunopathological feature of COVID-19 and it has been associated with the severity of the disease. The increase in blood concentrations of different cytokines such as interleukins and chemokines such as IL-6, IL-8, IL-10, TNF-α, IL-1β, IL-2, IP-10, MCP-1, CCL3, CCL4, and CCL5 has been described for COVID-19 patients (4). Some of these molecules have been proposed as biomarkers to monitor the clinical evolution and to determine treatment selection for COVID-19 patients (21–23). Nevertheless, it is important to consider that some of these molecules function in a context dependent manner, therefore the clinical relevance of analyzing single cytokine changes is limited.

One of the most important challenges during the pandemics is to avoid the saturation of the health systems, therefore the determination of predictive biomarkers that allow a better stratification of the patients is paramount. Even though cytokines such as IL-6 and IL-8 have been proposed as indicators of the disease severity, and in some studies they were strong and independent predictors of patient survival (24), their predictive value when analyzed alone is debatable (24). The generation of scores considering blood levels of cytokines such as interleukins and chemokines with different immunological functions incorporates the importance of the context-dependent function of these molecules.

In order to predict Severe cases, a score was generated considering blood concentrations of inflammation-associated factors such as IL-10, IL-6, IL-2, and IL-8, as well as sCD40L and VEGF which are associated with vascular homeostasis (25, 26). In this classification, Severe cases are characterized by high IL-6 and IL-10 levels, both cytokines previously attributed to increase the immunopathogenesis of COVID-19 and predictive value in Severe cases (22, 23). In different backgrounds, IL-6 has been associated with oxidative stress, inflammation, endothelial dysfunction, and thrombogenesis (25–28) which are characteristic features of Severe COVID-19 cases caused by excessive myeloid cell activation (29). Consistently, increased IL-10 levels interfere with appropriate T-cell responses, inducing T-cell exhaustion and regulatory T cell polarization leading to an evasion of the antiviral immune response (30). Furthermore, besides its anti-inflammatory function on T cells, in some backgrounds IL-10 induces STAT1 activation and a pro-inflammatory response in type I IFN-primed myeloid cells (30, 31). Therefore, elevated levels of IL-6 and IL-10 promote myeloid cell activation, oxidative stress, endothelial damage, which might affect an adequate antiviral T cell activation (26–30).

Furthermore, Severe cases show high levels of sCD40L and VEGF, which are associated with vasculitis and vascular remodeling. The cytokine storm observed in SARS-CoV-2 infection is accompanied by hemostatic alterations and thrombosis. sCD40L is a platelet activation marker, which has been associated with increase severity in COVID-19 patients (32–34). Moreover, sCD40L levels are higher in male patients compared with females and it is the sex-associated differences in the severity of the disease (33). Another vascular alteration associated to SARS-CoV-2 infection is endothelial hyperactivation. According to the proposed severity score, VEGF levels were significantly elevated in hospitalized COVID-19 patients when compared to Mild-Moderate cases. Additionally, to strengthen the classification presented here, the score differentiates the Severe cases by the denominator of IL-2 and IL-8, which are cytokines related to proper T cell activation (IL-2) and recruitment (IL-8) (35, 36).

According to the score generated for distinguishing PASC, these patients are characterized by an increased IFN-γ and IL-2 and a reduced CCL4 production. In the context of a viral infection, the combination of IFN-γ and IL-2 would induce the activation of effector T cells with pro-inflammatory properties and the capacity of generating an effective immune response to eliminate the virus. However, PASC are characterized by longer periods of time with clinical signs and symptoms such as fatigue and lung damage. This suggests that the inflammatory context created by these cytokines that leads to T cell activation is not enough to generate an adequate anti-viral response without the proper recruitment signals to attract activated T cells. CCL4 signals through the receptor CCR5 to attract T cells to the site of inflammation and depending on the immune context, this molecule recruits differently activated T cells (37, 38). Moreover, it was recently shown, by single cell analysis, down regulation of CCL4 expression in peripheral myeloid cell compartments in patients with Mild and Severe COVID-19 (39). In PASC, IFN-γ and IL-2 would create an immune context favoring the Th1 polarization, but the low levels of CCL4 affect the recruitment of these cells thus impairing the antiviral response should SARS-CoV-2 RNA or protein persist. The effect of increased IFN-γ and IL-2 on T cell activation is evident in the reduction of the frequency of exhausted (CD4+PD1+/CD8+PD1+) and total regulatory T cells (FoxP3+) compared to healthy donors. Therefore, proper T cell activation (high IFN-γ+IL-2) but ineffective T cell recruitment (low CCL4) are characteristic features of the failed anti-viral response observed in the PASC group supporting virus persistence. 

The significant increase of B cells in the PASC group is associated with high IL-2 levels promoting B cell proliferation and differentiation (40). Interestingly, increased IFN-γ affects B-cell homing to lymph nodes (41), reduces total IgG production, and inhibits pre-activated B cells (42). This could be associated with virus persistence in the PASC group as supported by the low CCL4 levels observed in these patients, since CCL4 has been proposed as a biomarker for B cell receptor pathway activation (43).

Additionally, increased IFN-γ promotes myeloid cell activation which is observed in the augmented frequency of inflammatory CD14+, CD16+, CCR5+ monocytes in the PASC group compared to healthy donors, supporting lymphopenia and virus persistence in these patients. This is in line with recent findings describing increased gene expression in response to IFN-γ in Mild and Severe COVID-19 patients in peripheral myeloid cells (39) and the dysregulation in the balance of monocyte populations by the expansion of the monocyte subsets described in COVID-19 patients (39). Finally, we propose that long-lasting pulmonary damage observed in PASC, is caused by a combination of factors including 1) virus persistence influenced by the PASC immune profile as characterized by high IFN-γ and IL-2 levels. This in turn induces Th1 polarization which is ineffective with low CCL4-induced T cell recruitment, leading to an inflammatory myeloid cell activation; and 2) the immunopathological pulmonary effects of this PASC immune profile. Regarding the immunopathological effects of the PASC immune profile, it has been shown using murine models that high IFN-γ levels could affect the kinetics of the resolution of inflammation-induced lung injury as well as thrombus resolution (44–46), which could be related to long-lasting symptoms of PASC associated to pulmonary coagulopathy and immune-mediated tissue damage.

Interestingly, COVID-19 individuals (including PASC, Mild, Severe) show high levels of CCL5, a chemokine that like CCL4 signals through CCR5. Indeed, the disruption of the CCL5-CCR5 pathway restores immune balance in critical COVID-19 patients (4). In the specific case of PASC, despite the statistically significant elevation of CCL5 compared to healthy controls, a reduction in the CCL4-mediated recruitment of activated T cells is proposed. This could be related to different factors:

(1) Reduction of total recruitment signals in PASC with low CCL4 concentrations.
(2) Different functional responses of CCL4 and CCL5 to polymorphic variants of the CCR5 gene. Distinct functional features have been reported in CCR5 variants regarding binding avidity, receptor internalization, Ca++ influx and chemotactic activity (47). Even though, clear mechanistic differences between CCL4 and CCL5 interaction with CCR5 are missing, even considering the knowledge gained on CCR5 polymorphisms in HIV/AIDS context (48).
(3) Signaling through alternative receptors for CCL5. Besides CCR5, CCL5 can signal through the receptors CCR1 and CCR3 (49) whereas CCL4 effects are restricted to CCR5. It has been shown that CCL4 can bind to CCR1 but is not able to induce the intracellular pathway necessary for activating the chemoattractant stimulus (49). Therefore, CCL4 has been proposed as an antagonist of CCR1 (50), however further analysis of this needs to be performed. Interestingly, CCR1 is expressed on blood myeloid cells such as monocytes and neutrophils, and it is upregulated on COVID-19 patients (51). Additionally, high levels of IFN-γ (a feature of PASC) have been associated with an increase in CCR1 expression on human neutrophils (52). Therefore, in PASC, high levels of CCL5 (combined with low levels of potential CCR1-antagonist CCL4) leads to a higher recruitment of myeloid cells expressing CCR1.

## Conclusion

In conclusion, we developed a bioinformatics pipeline that analyzed cytokines of the immunological landscape of COVID-19 using machine learning methods to discriminate between PASC and Severe individuals from other classes. The implementation of random forest classifiers allowed for the identification of the critical cytokines for this discrimination, which in turn was used to calculate highly sensitive heuristics for PASC and Severe individuals. These models, which can be incorporated into clinical laboratory information systems, enabled a highly accurate, immune-based classification of severe COVID-19 infection and PASC. This workflow could greatly aid the triage, treatment, and prognosis of those affected. An interesting caveat affecting the specificity of the PASC classification was that 7 Severe COVID-19 patients classified as PASC that, while affecting the specificity of PASC classification, may represent a subset of acute COVID-19 patients destined to become affected by PASC.

These data also indicate that with an effective classification of severe and PASC individuals based on cytokine profiles, precision therapies guided by the machine learning output may result in lower severity and PASC scores and possibly in more favorable clinical outcomes. CCR5 antagonism has already been demonstrated to reduce IL-6, and VEGF (4, 53), numerators in the severity score, and to reduce IFN-γ, a numerator in the PASC score (54).

## Data Availability Statement

The original contributions presented in the study are included in the article/**Supplementary Material**. Further inquiries can be directed to the corresponding author.

## Ethics Statement

Informed consent was obtained from all participants. Samples were considered exempt for the purposes of this study and results were not used to manage patients. The patients/participants provided their written informed consent to participate in this study.

## Author Contributions

RY organized the clinical study and actively recruited patients. BP, AP, HR, and EL performed experiments and analyzed the data. JG-C, RM-R, and JM performed the bioinformatics. BP, JM, JG-C, RM-R wrote the draft of the manuscript. All authors contributed to the article and approved the submitted version.

## Conflict of Interest

BP, AP, HR, and EL are employees of IncellDx.

The remaining authors declare that the research was conducted in the absence of any commercial or financial relationships that could be construed as a potential conflict of interest.

## Abbreviations

IL, interleukin; RANTES, regulation on activation, healthy control T-expressed and secreted; CCR, chemokine receptor; IFN, interferon; TNF, tumor necrosis factor; MIP, macrophage inflammatory protein; GM-CSF, granulocyte-macrophage colony-stimulating factor; VEGF, vascular endothelial growth factor; HIV, human immunodeficiency virus; HCV, hepatitis C virus.
