# Supplementary material for: Immune-Based Prediction of COVID-19 Severity and Chronicity Decoded Using Machine Learning
Source: Front Immunol. 2021 Jun 28;12:700782. doi: 10.3389/fimmu.2021.700782 (PMC8273732; doi:10.3389/fimmu.2021.700782)

## Slide 1
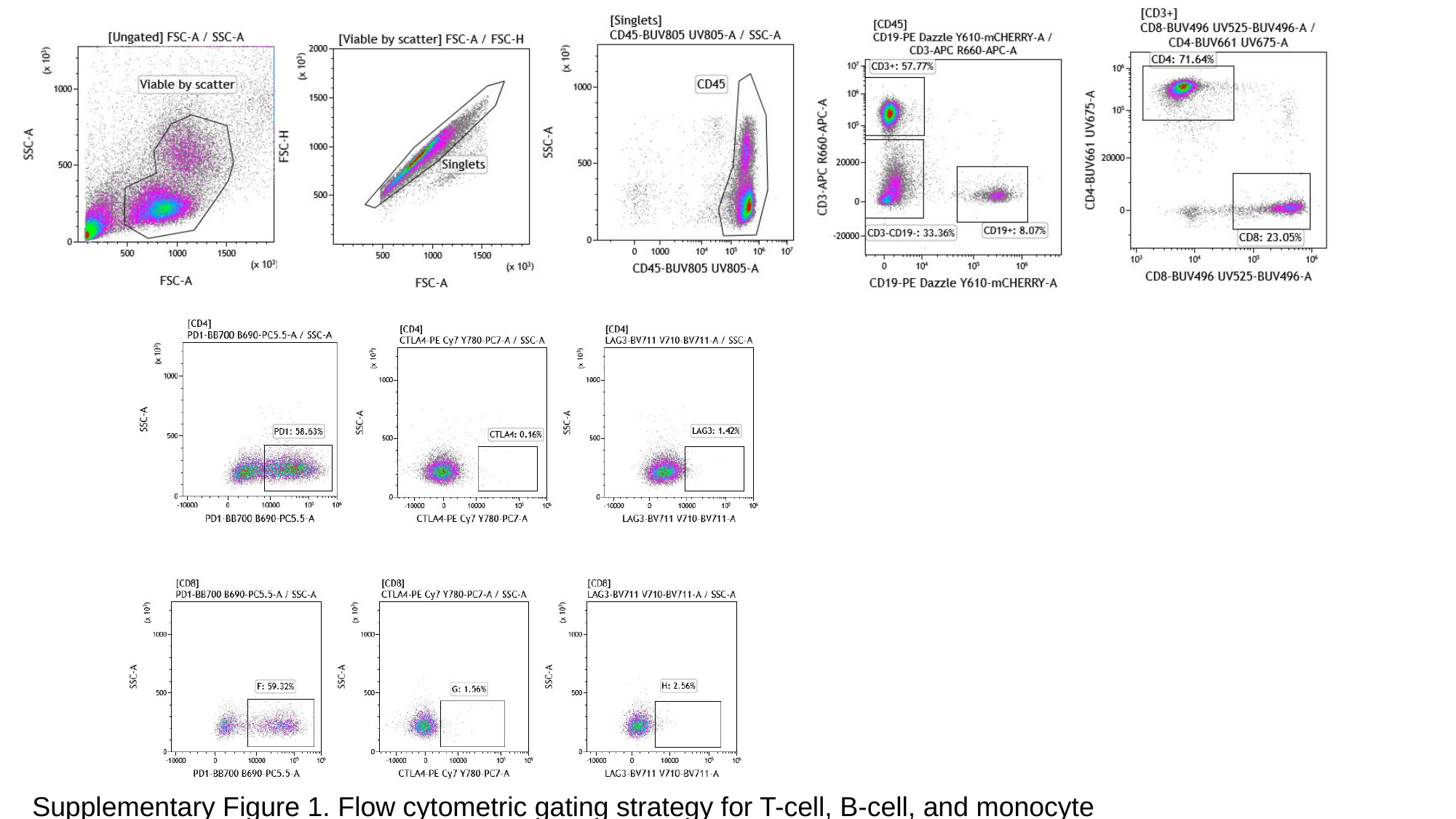

Supplementary Figure 1. Flow cytometric gating strategy for T-cell, B-cell, and monocyte analysis

## Slide 2
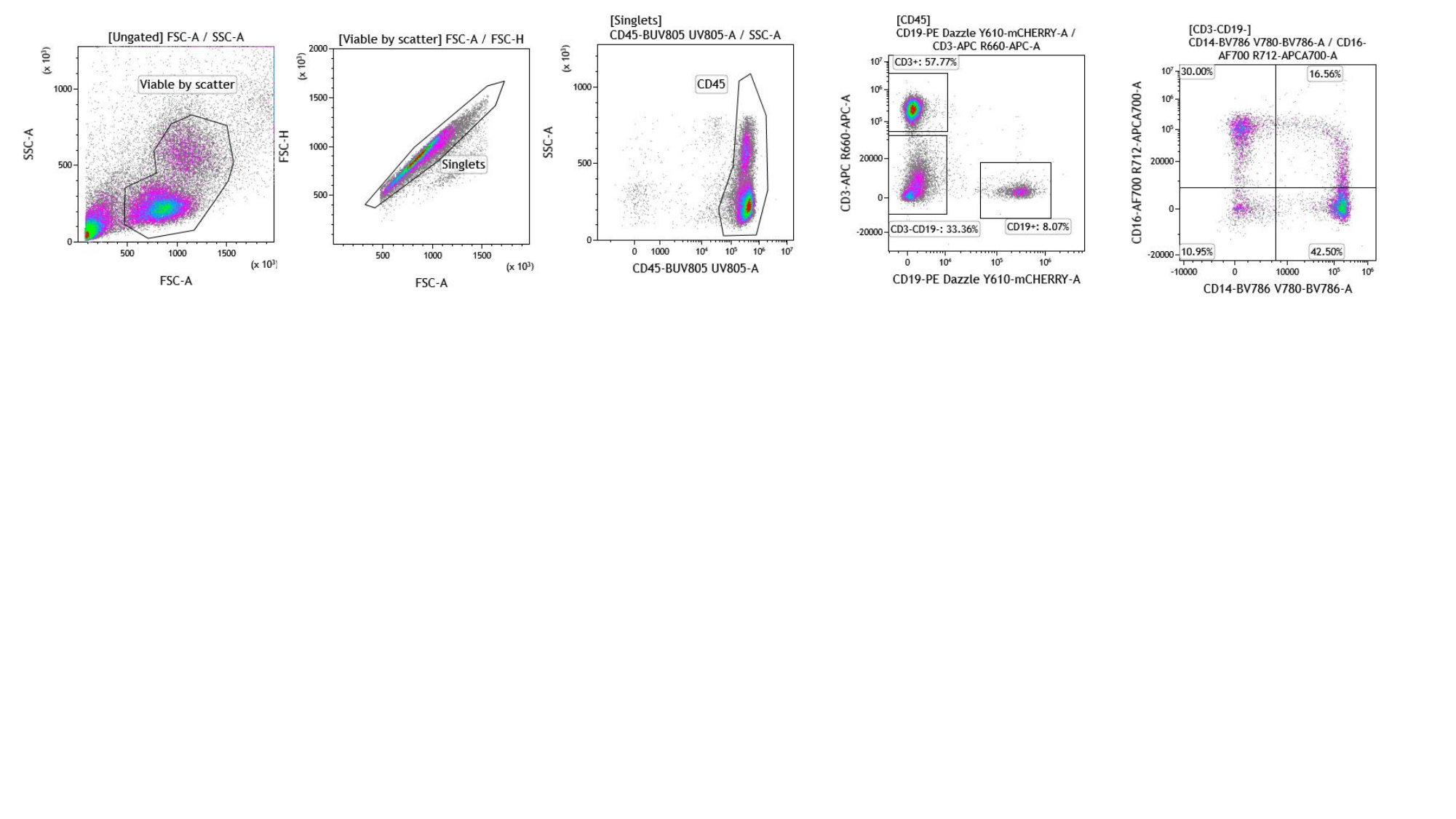

Supplement: Supplementary file 1 [file Presentation_1.pptx]
