# Supplementary material for: Immune-Based Prediction of COVID-19 Severity and Chronicity Decoded Using Machine Learning
Source: Front Immunol. 2021 Jun 28;12:700782. doi: 10.3389/fimmu.2021.700782 (PMC8273732; doi:10.3389/fimmu.2021.700782)
Supplement: Supplementary file 2 [file Table_1.docx]

| **Immune Profiling Markers** | **luorochrom** | **Clone** | **Vendor** | **µg per Test** |
| --- | --- | --- | --- | --- |
| CD8 | BUV496 | RPA-T8 | BD | 0.2 |
| CD4 | BUV661 | SK3 | BD | 0.08 |
| CD45 | BUV805 | HI30 | BD | 0.1 |
| CD103 | BV421 | Ber-ACT | BioLegend | 0.2 |
| TIM3 | BV605 | 7D3 | BD | 0.6 |
| CD56 | BV650 | HCD56 | BioLegend | 0.3 |
| CD14 | BV786 | M5E2 | BioLegend | 0.3 |
| PD-1 | BB700 | EH12.1 | BD | 0.6 |
| FoxP3 | PE | 259D/C7 | BD | 0.3 |
| CD19 | PE-Dazzle5 | HIB19 | BioLegend | 0.15 |
| CD3 | APC | UCHT1 | BioLegend | 0.05 |
| CTLA-4 | PE-Cy7 | BN13 | BioLegend | 0.3 |
| CD16 | AF700 | 3G8 | BioLegend | 0.75 |
| HLA-DR | APC/Fire750 | L243 | BioLegend | 0.6 |

Supplementary Table 1. Antibody clones, labels, and amounts used in T-cell, B-cell, and Monocyte analysis
